# The innate defenders: a review of natural killer cell immunotherapies in cancer

**DOI:** 10.3389/fimmu.2024.1482807

**Published:** 2024-12-23

**Authors:** Pablo Álvarez-Carrasco, Carmen Maldonado-Bernal

**Affiliations:** ^1^ Laboratorio de Investigación en Inmunología y Proteómica, Hospital Infantil de México Federico Gómez, Mexico City, Mexico; ^2^ Facultad de Medicina, Universidad Nacional Autónoma de México, Mexico City, Mexico

**Keywords:** natural killer cells, cancer, immunotherapy, activating signals, inhibitory signals, CAR-NK cells

## Abstract

Cancer is a condition that has been with us for centuries; however, the therapies that have been developed are often associated with significant toxicity and various side effects. Recent advances in immunology have revealed the potential of the immune system to fight cancer, leading to the emergence of immunotherapy. This review focuses on Natural Killer (NK) cells, innate immune effectors with a remarkable ability to directly kill cancer cells. We will explore the historical context of cancer treatment, the nature of NK cells, and the ways they have been developed to enhance their anti-tumor function, highlighting the limitations of conventional therapies. The therapeutic potential of NK cell-based immunotherapies will also be discussed, emphasizing their unique advantages over other immune cell-based approaches. This review highlights the promising future of NK cell therapies in the fight against cancer and their possible application to assist and improve conventional therapies developed so far.

## 1 Introduction

Cancer, a well-documented disease, has likely afflicted humans throughout history. The oldest records of this terrible disease date back to the time of the Egyptians, in the Edwin Smith papyrus (written around 3000 BC). Here, they documented cases of tumors and breast lumps, a grave condition with a single, stark verdict: “no treatment” (1).

For centuries, the only way to treat cancer was through surgery, which often proved ineffective. In the 20th century, radiotherapy and chemotherapy significantly advanced cancer treatment. These therapies target rapidly dividing cancer cells by interfering with their cell cycle. However, their effectiveness is limited by their toxicity to healthy cells, which also divide through mitosis. This can lead to severe side effects and even the development of secondary cancers (2, 3).

Advances in immunology have not only shown us the complex response that leukocytes exhibit in recognizing and eliminating viruses, bacteria, fungi, and other external agents that can be harmful to our body, but also their extraordinary ability to recognize and eliminate transformed cells.

This review highlights the paradigm shift from traditional therapies to the growing field of immunotherapy. It underscores the immense potential of manipulating the immune system to combat cancer. In particular, the exciting possibilities offered by NK cell-based immunotherapies are a testament to this potential. This paves the way for a deeper exploration of specific immunotherapy approaches within the academic discourse.

## 2 Natural killer cells: innate defenders against cancer

Fueled by advancements in immunology, scientists began to unravel the intricate workings of leukocytes, particularly their remarkable ability to recognize and eliminate not only invading pathogens but also transformed cells within the body. This newfound understanding paved the way for the concept of immunological surveillance of cancer, proposed by Lewis Thomas and Frank Macfarlane Burnet over five decades ago (4). This concept essentially posits that the immune system has a built-in capacity to identify and eliminate cancerous cells.

This revelation spurred the development of diverse and promising immunotherapy approaches that capitalize on the immune system’s inherent ability to combat cancer. One such example is the use of monoclonal antibodies. These sophisticated tools can either block inhibitory signals that would otherwise restrain cytotoxic cells from attacking cancer or stimulate effector cells like NK cells recruiting them through a process called antibody-dependent cellular cytotoxicity (ADCC), leading to the destruction of cancer cells (5, 6).

However, the potential of immunotherapy extends beyond just this approach. Natural Killer (NK) cells, for instance, hold promise. These cells can directly recognize and eliminate cancer cells without prior sensitization. This unique characteristic makes NK cells ideal candidates for adoptive cell therapy, in which a patient’s own NK cells are expanded and reintroduced to target their tumors (7–10).

Human NK cells can be broadly classified into two subsets based on CD56 and CD16 surface marker expression: CD56^bright^CD16^dim^/low, these cells are less cytotoxic but secrete pro-inflammatory cytokines upon stimulation (7, 11) CD56^dim^CD16^high^; this highly cytotoxic subset dominates the peripheral blood and plays a major role in NK-mediated tumor cell killing (7, 11).

However, it is still necessary to delve deeper into this field. One of the newest analyzes carried out to determine the distribution and characteristics of NK cells is that of Rebuffet et al.*, 2024.* They identified three primary populations of natural killer (NK) cells, designated as NK1, NK2, and NK3, using a combination of single-cell RNA sequencing (scRNA-seq) and cellular indexing of transcriptomes and epitopes by sequencing (CITE-seq). This advanced technique allowed for a detailed analysis of the gene expression profiles and protein markers of individual NK cells, enabling their classification into distinct subsets:

NK1 cells are characterized by their robust cytotoxic effector functions, making them highly effective at killing target cells. They are primarily found in the bloodstream and constitute approximately 60% of circulating NK cells.

NK2 cells are linked to chemotaxis regulation and leukocyte differentiation, suggesting they play a role in guiding other immune cells to the site of an infection or tumor. They are less abundant than NK1 cells, representing about 17% of circulating NK cells.

NK3 cells are known for their upregulation in leukocyte activation, indicating their potential role in activating other immune cells. They are enriched in HCMV-positive individuals and comprise about 24% of circulating NK cells (12).

Research such as this has significantly deepened our understanding of the intricate complexities of the cellular immune response. However, further exploration is necessary to translate these insights into tangible improvements in treatments for a wide range of conditions.

NK cells form part of the innate lymphoid cell (ILCs) family. They are critical components of the innate immune system, recognizing and eliminating virus-infected or malignant cells. Unlike T cells, which recognize specific antigens presented by antigen-presenting cells (APCs), NK cells rely on a “missing-self” or “stress-induced self” recognition mechanism to identify and eliminate abnormal cells. This recognition occurs through a delicate balance of activating and inhibitory signals received by NK cells via cell surface receptors and the fate of both. The NK cell, acting as a sentinel, assesses this balance to determine the fate of the target cell: whether it lives or dies (7, 10).

### 2.1 Dual recognition system

#### 2.1.1 Activating signals

Cancer cells often exhibit changes in their surface molecules. These changes can include the downregulation of HLA class I molecules (missing-self recognition) or the upregulation of stress ligands, such as MHC class I-related chain A (MICA) and MICB. NK cells express receptors that recognize stress-induced ligands upregulated on transformed cells. Key activating receptors include NKG2D (13), DNAM-1 (14), and NKp46 (15). The ligands of this receptors, such as MICA/B (16) and ULBPs (16, 17), are normally absent or expressed at low levels on healthy cells but become prominent on stressed or cancerous cells (18).

NKG2D exemplifies a crucial activating receptor for NK cells. Its ligands (e.g. MICA/B) are upregulated on stressed or transformed cells, allowing NK cells to recognize and eliminate these potential threats. Studies suggest that oncogenes can directly induce the NKG2D ligand expression on cancer cells, highlighting its role in cancer immunosurveillance (16, 17).

#### 2.1.2 Inhibitory signals

NK cells also possess inhibitory receptors that recognize self-major histocompatibility complex class I (MHC-I) molecules on healthy cells. This “missing-self” recognition prevents NK cells from attacking healthy tissues. Mainly, the so called KIRs (Killer Immunoglobulin Receptors) mediate the recognition of “self”. Their family includes activating and inhibitory receptors, and both are an extremely polymorphic family (19).

NKG2A exemplifies an inhibitory receptor containing an ITIM cytoplasmic domain. After binding with specific haplotypes of Human Leukocyte Antigen class 1 molecules (specifically with HLA-E), this receptor exerts its inhibitory function signaling through SHP-1/2, which can dephosphorylate multiple targets in the ITAM-activating pathway (20). NK cell inhibition is essential to prevent their attacks on healthy ‘self’tissue. Cancer cells that downregulate MHC-I to evade T cells become susceptible to recognition and attack of NK cells (19).

The balance between activating and inhibitory signals received by NK cells plays a crucial role in regulating their cytotoxicity but there are several factors that can influence this balance, such as immunomodulatory factors. Cytokines such as interleukin (IL)-2, IL-12, and IL-15 can activate and stimulate NK cell proliferation and cytotoxicity. On the other hand, cytokines such as transforming growth factor-beta (TGF-β) can suppress NK cell activity (21–23).

**Figure 1:**
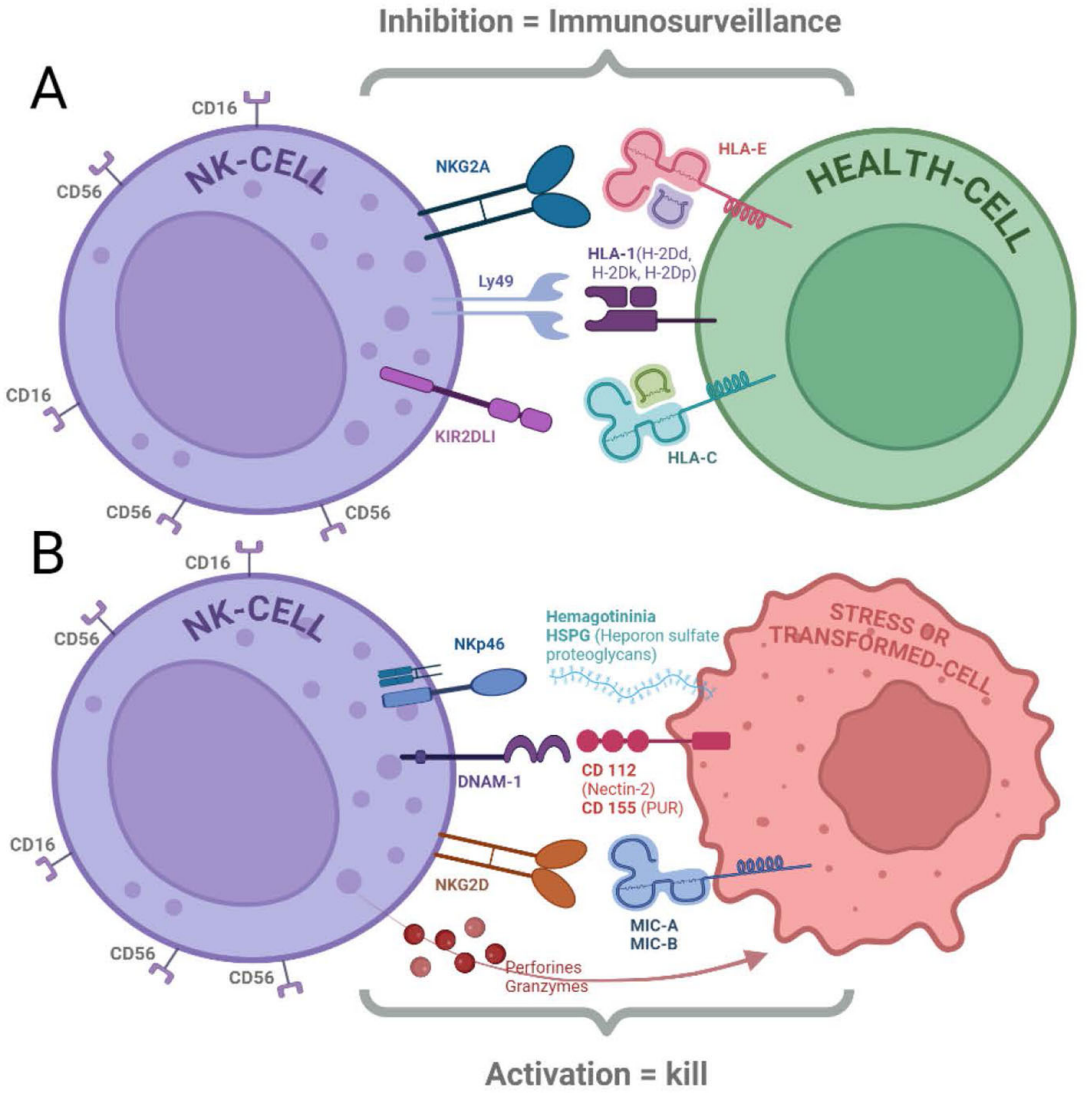
NK cells express receptors that regulate their cytolytic function. **(A)** Inhibitory Receptors: Binding of ligands to inhibitory receptors (NKG2A, Ly49, and KIR2DL1) can suppress NK cell cytotoxicity. These receptors primarily recognize HLA molecules expressed on healthy cells (18, 20–22). **(B)** Activating Receptors: NK cells also possess activating receptors (NKp46, DNAM-1, and NKG2D) that trigger cytotoxicity when engaged with their ligands (HSPG, CD112/CD155, and MIC-A/B, respectively). Cytolytic activity is initiated by both the activation of these receptors and the absence of inhibitory receptor signaling, leading to the release of perforins and granzymes (13, 15, 16, 18).

The tumor Microenvironment is another important factor that must be considered. Tumors often create an immunosuppressive microenvironment rich in inhibitory factors and are depleted of activating signals. This can significantly suppress NK cell activity and hinder their effectiveness in eliminating cancer cells (24–26) **Figure 1**.

### 2.2 Direct cytotoxicity and elimination of cancer cells

At this point, we have discussed that NK cells are a vital component of the innate immune system, playing a central role in immune surveillance and the elimination of abnormal or infected cells, including cancer cells. Their ability to directly kill target cells, particularly tumors, through a process called cytolysis, makes them a potent weapon against cancer. Here is a deeper look into the mechanisms of NK cell-mediated direct cytotoxicity and the elimination of cancer cells:

**Granule Exocytosis.** NK cells harbor specialized cytoplasmic granules filled with perforin and granzymes. When an NK cell encounters a target cell, these granules are released towards the target cell membrane. Perforin, a pore-forming protein, creates perforations in the target cell membrane, allowing the entry of granzymes. The process includes granule convergence, polarization towards the immunological synapse and subsequent exocytosis. Once inside the target cell, granzymes, a family of serine proteases, trigger the execution phase of apoptosis (programmed cell death) by cleaving vital cellular proteins (27, 28).

**Death Receptor Ligation.** NK cells express Fas ligand (FasL), a molecule that can bind to Fas death receptors present on the surface of target cells. This binding interaction initiates a signaling cascade within the target cell, ultimately leading to the activation of caspases, a group of enzymes responsible for dismantling cellular components and inducing apoptosis (21).

### 2.3 Immunomodulatory effects and activation of other immune cells

NK cells are well-recognized for their direct cytolytic activity against tumor cells and infected cells. However, their role in the immune system extends far beyond this. NK cells express Toll-like receptors (TLRs), which act as crucial sensors for recognizing pathogens and alarm signs and for initiating innate immune responses (29, 30). This section explores the intricate interplay between NK cells and TLRs, studying how they influence both, the function of NK cells themselves and the activation of other immune cells.

Initial studies identified TLR expression in NK cells through mRNA detection. Human NK cells isolated from peripheral blood constitutively express mRNA for TLRs 1-8, with TLR2 and 3 being the most abundantly expressed. However, the expression profile can vary; TLR2 mRNA is highly expressed, TLR4 and TLR3 mRNAs are weakly expressed, and TLR8 mRNA might not be detectable in resting human NK cells. Additionally, NK cells more frequently express TLRs 2, 3, 5, and 6 compared to TLRs 4, 7, 8, and 9 (31). A study was recently published showing the expression of the 10 TLRs as protein in cytotoxic NK cells from healthy children and children with acute lymphoblastic leukemia (ALL) (32).

Activation of TLRs on NK cells triggers diverse responses. For instance, TLR2 activation using lipophosphoglycan increases IFN-γ levels and cell surface TLR2 expression. Poly-inosinic-cytidylic acid [poly (I:C)] activates human NK cells via TLR3, while TLR9 activation induces human NK cells to secrete IFN-γ and TNF-α. Several studies have shown that TLR stimulation leads to the secretion of IFN-γ by human NK cells (31) Beyond their direct effects, NK cells can influence other immune cells through TLR-mediated mechanisms.

Additionally, diseases like acute lymphoblastic leukemia (ALL) could affect NK cell populations and TLR expression. Research by Sánchez-Herrera et al. (2021) suggests a decrease in NK cell number and altered TLR expression in pediatric ALL patients (32). Their findings showed that cytotoxic NK cells from children with ALL expressed all 10 TLRs, but with decreased expression of TLR1 and TLR9 compared to healthy controls. Interestingly, the study also revealed the first evidence of TLR10 expression in cytotoxic NK cells from ALL patients. The different TLRs showed alterations in expression levels and cellular distribution, suggesting a potential role in the immune response to leukemia. In a recent study of NK cells obtained from patients with ALL, it was observed that the ligands of TLR3 (Poly I:C) and TLR7 (Imiquimod) increased the activation of NK cells, increasing the expression of IFN-γ, CD107a, NKG2D, and NKp44, and very importantly, the ligands of TLR8 (R848) and TLR9 (ODN 2006) increased cytotoxicity against leukemic cells (33).

Given the immunomodulatory effects of TLRs on NK cells, researchers are exploring their potential for cancer immunotherapy:

Schölch et al., 2015, highlight the promise of combining TLR agonists with radiotherapy. This approach activates various immune cells, including NK cells, CD8+ T cells, and dendritic cells, leading to a more robust anti-tumor response against gastrointestinal tumors, such as colorectal and pancreatic cancers. The combined therapy improves both local and distant tumor control, addressing the challenges of treating metastatic tumors (34).

Park et al., 2018, studied the use of TLR agonists (R848 as TLR7/8 agonist) delivered via a hydrogel scaffold for perioperative immunotherapy. Their findings suggest this approach can promote antitumor immune responses, prevent tumor recurrence, and eliminate metastases in different cancers. The study highlights the importance of the local and extended release of TLR agonists from the scaffold for therapeutic benefit. It also explores the impact on specific immune cell subsets like NK cells, CD8+ T cells, and CD4+ T cells, along with cytokine production, immune cell proliferation, and induction of antitumor memory responses. Notably, in NK cells, this resulted in an increase in the number of activated NK cells, contributing to the prevention of local tumor recurrence and eradication of existing metastases (35).

Targeting Specific NK Cell Subsets, Veneziani et al. (2022) focused on the response of CD56^bright^CD16- NK cells to TLR7/8 agonists. The researchers found that R848, a TLR7/8 agonist, delivered the strongest signal to NK cells, primarily activating the CD56^bright^CD16- NK cell subset. R848 selectively increased the proliferation, cytokine production, and cytotoxic activity of CD56^bright^CD16- NK cells, predominantly via TLR8. In contrast, other TLR agonists, such as Poly I:C and ODN2395 did not induce similar responses. The study also demonstrated that TLR8 agonists potentiate chemokine production, increase cytotoxicity, and can synergize with other TLR agonists on NK cells. Their study suggests that TLR8 agonists may be a valuable target for NK cell-based immunotherapy in cancers like metastatic ovarian (30).

## 3 Enhancing NK cell function

### 3.1 Sourcing and expanding NK cells

Cancer therapies are constantly evolving, and NK cell infusions are emerging as a powerful contender. These infusions leverage the body’s natural defense system by harnessing the ability of NK cells to destroy cancer cells. Obtaining NK cells for infusions can be either by extracting them from a patient’s own blood (autologous) or from a donor (allogeneic). Researchers are also developing methods to expand these cells in the laboratory, potentially creating a more readily available source for treatment. This ability to source and amplify NK cells is crucial for making this therapy a more viable and impactful approach in the fight against cancer (8, 36).

#### 3.1.1 *Ex vivo* expansion and activation techniques

NK cells constitute 10-20% of peripheral blood mononuclear cells (PBMC). Despite not being very abundant, there exist several protocols to isolate and expand these primary NK cells from PBMC for therapeutic purposes (8, 37).

Early clinical trials in the 1980s used stimulating factors, such as interleukin-2 (IL-2) to enhance NK cell potency *in vivo*. However, these resulted in poor outcomes due to a high IL-2 toxicity. Approaches like generating lymphokine-activated killer (LAK) cells through *in vitro* IL-2 stimulation also yielded limited success (8). High-dose IL-2 administration caused severe side effects and stimulated regulatory T cells, further hindering this approach. However, recent studies have demonstrated that low-dose IL-2 therapy can selectively expand NK cell populations *in vivo* (38). Additionally, IL-2 alone can induce the proliferation of NK cells *in vitro* (39–41).

Current strategies recognize that NK cell expansion requires multiple signals for survival, proliferation, and activation. Researchers are focusing on replacing these factors using autologous feeder cells or genetically modified allogeneic feeder cells. Examples include the Jurkat KL-1 T lymphoblast subline and the genetically modified K562 feeder cells engineered to express membrane-bound IL-15 and CD137L (8).

The unique properties of NK cells discussed earlier highlight their potential as targeted therapy for cancers. However, due to their limited numbers among lymphocytes, *ex vivo* expansion of NK cells before infusion is crucial to maximize the potency of NK cell therapies.

Expanding NK cells is not an easy task. Studies have shown that simply exposing NK cells to cytokines such as IL-2 is not sufficient for sustained proliferation. While IL-2 can stimulate some growth, it lacks the potency and durability needed for a robust expansion without additional stimuli (42). Furthermore, NK cell proliferation has been attempted with a large number of factors. Below we mention some that have been carried out successfully:

Recent research by Wang et al., 2024, demonstrates the *ex vivo* expansion and activation of NK cells for bladder cancer treatment. Their method involves culturing PBMCs from healthy donors in a medium containing growth factor such as IL-15, IL-2, and OK432 (43). OK432 is a preparation made from a low-virulence strain of *Streptococcus pyogenes*. It has been used as an immunotherapeutic agent for the treatment of various cancers, including bladder cancer (44). This combination promotes NK cell growth and expansion.

Studies by Denman et al., 2012, and Li et al., 2015, explored the effects of IL-21 on NK cells during *ex vivo* expansion. While IL-21 increases NK cell cytotoxicity and proliferation in a controlled dose, high concentrations can shorten their lifespan by inducing cell death (apoptosis). This highlights the importance of optimizing the IL-21 dosage for NK cell expansion (45, 46).

The research by Fernández et al., 2021, investigated methods to optimize NK cell expansion and activation for clinical use. They identified NK MACS and TexMACS media as superior for achieving a high NK cell purity and a low T cell contamination. Additionally, the study revealed that PBMC yielded a higher number of expanded NK cells compared to CD45RA+ cells, although NK purity was similar. Notably, using PBMC and K562mbIL21-41BBL cells achieved the highest fold expansion and NK cell purity. This research provides valuable insights into optimizing NK cell culture conditions and activation strategies for large-scale clinical manufacturing (47).

Effective NK cell expansion methods involve co-culturing them with stimulatory cells. Examples include EBV-transformed lymphoblastoid cells, Wilms tumor cell lines, or leukemia cell lysates. These stimulatory cells provide the necessary signals for NK cell proliferation and activation (8, 48, 49).

Szmania et al., 2015, investigated the use of genetically modified feeder cells for NK cell expansion. They used an engineered K562 cell line expressing the membrane-bound IL-15 and 41BB-ligand. This approach resulted in robust *in vivo* expansion of NK cells generated from myeloma patients and haploidentical donors, particularly with higher IL-2 concentrations (500 U/mL) in the culture medium (50).

Another approach includes bioreactor expansion. Schlegel et al., 2019, compared static expansion (culturing cells in flasks) with a semi-automated approach using a bioreactor. Interestingly, the static method yielded significantly higher NK cell numbers compared to the bioreactor approach. This suggests that static culturing might be more suitable for large-scale clinical-grade *ex vivo* expansion and activation of NK cells for therapeutic applications (49).

Studies such as those by Schlegel et al., 2019, demonstrate the promise of *ex vivo* expanded and preactivated NK cells for cancer treatment. The expanded cells, especially those with a CD56^bright^CD69^high^ immunophenotype, demonstrated excellent direct cellular cytotoxicity and antibody-dependent cellular cytotoxicity (ADCC) against various tumor entities, resulting in a significant increase in direct antitumor activity and the reduction of minimal residual disease (MRD) post cell transfer. The results supported the potential for clinical testing of expanded NK/γδT/CIK cells for cancer therapy (49).

Another approach to enhance cytotoxicity is the indirect activation through plasmacytoid Dendritic Cells (pDC) and its TLRs. In the study by Belounis et al., 2020, they demonstrated that TLR-activated pDCs can enhance the cytotoxic function of NK cells in high-risk neuroblastoma (NB) patients undergoing immunotherapy with dinutuximab. They found that TLR-activated pDCs increase the expression of TRAIL on NK cells, leading to increased cytotoxicity against patient-derived NB cells. It was found that patients displayed normal blood NK cell counts, but with a high proportion of CD56^bright^ CD16^low^/− cells, which were responsive to pDC stimulation. This suggests the potential of using TLR-activated pDCs in adoptive immunotherapy to decrease relapse risk in high-risk NB patients (51).

#### 3.1.2 Applications of expanded NK cells

Following *ex vivo* expansion and activation, NK cells with enhanced cytotoxicity and cytokine secretion can be used for various applications:

*In vitro*, in cytotoxicity assays, testing the effectiveness of NK cells against cancer cell lines. In patient-derived organoid models, evaluating NK cell efficacy in a more realistic 3D tumor microenvironment. *In vivo*, in xenograft mouse models, assessing NK cell efficacy in living animals with transplanted tumors.

*Ex vivo* expansion is a critical step in preparing NK cells for potential therapeutic applications against cancer. By optimizing these techniques and using them together with other advancements in NK cell therapy, researchers are paving the way for a new generation of cancer treatments.

### 3.2 Current treatments with NK cells

Autologous treatments may have a lower risk of rejection but may not be suitable for all patients due to limitations in the availability or quality of their own cells. Allogeneic treatments may have greater availability and diversity but require matching and immunosuppressive measures to reduce the risk of rejection (7, 52).

#### 3.2.1 Autologous NK cell therapy

Another promising approach is exemplified by a phase I clinical trial conducted in Vietnam (**Table 1**). This novel cancer treatment focused on expanding autologous NK cells and cytotoxic T lymphocytes (CTLs) for patients with lung, liver, and colon cancers. The study demonstrated a significant *ex vivo* expansion of NK cells and CTLs, with an average fold increase of over 500 after a three-week culture period. The therapy was associated with an improved quality of life, reduced fatigue, and a promising increase in mean survival time for the participating patients (55). These studies highlight the ongoing advancements in autologous NK cell therapy, offering a potentially powerful tool for cancer treatment.

**Table 1:** Clinical trials conducted with NK cells.

Author	Clinical Trial	Type of NK Cell Immunotherapy	Cancer Type	Main Results	Country
Liem NT, et al.	2517/BYT-KCB	Expansion of autologous T and NK cells	Colon, liver, and lung cancer	Improved quality of life and overall survival in patients with advanced-stage solid tumors.	Vietnam
Bednarski M, et al.	IRB#201709041	CAR-caspase-9 NK cells	CLL, non-Hodgkin lymphoma	Significant objective response rates, including complete and partial responses, in patients with relapsed/refractory B-cell malignancies.	United States
Liu E, et al.	NCT03056339	Infusion of *ex vivo* expanded autologous NK cells	Metastatic melanoma	Objective response rate of 20% in patients with metastatic melanoma, demonstrating the potential of NK cell therapy in solid tumors.	United States

#### 3.2.2 Allogenic NK cell therapy

Allogeneic NK cell therapy offers an alternative approach to address the limitations associated with autologous NK cells. These limitations include potential dysfunctionality of autologous NK cells in cancer patients and the challenges of engineering them to effectively recognize malignant cells (37).

A key aspect of allogeneic NK cell therapy is the use of KIR-mismatched NK cells from healthy donors. Killer cell immunoglobulin-like receptors (KIRs) are cell surface molecules on NK cells that can recognize specific human leukocyte antigen (HLA) molecules on target cells. KIR mismatch between donor and recipient can enhance the recognition and elimination of tumor cells by the infused NK cells (37). However, selecting the appropriate donor with a compatible KIR haplotype is crucial to optimize the effectiveness and reduce the risk of graft-versus-host disease (GVHD).

A potential concern with allogeneic cell therapy is GVHD, where the transplanted immune cells attack the recipient’s body. To minimize this risk, allogeneic NK cell products often undergo T-cell depletion. T cells are the primary immune cells responsible for GVHD, and their removal significantly reduces the risk of this complication (37).

A promising avenue in allogeneic NK cell therapy involves the use of memory-like NK cells. Unlike traditional NK cells, which are part of the innate immune system and lack an immunological memory, memory-like NK cells exhibit enhanced anti-tumor activity and can persist in the body for extended periods, similar to memory T cells from the adaptive immune system. This unique characteristic is highlighted by the term “memory-like” (56).

A recent study by Kulkarni et al., 2023, evaluated the feasibility of using autologous CD56-positive cells (enriched NK cells) from haploidentical family donors exposed to autologous plasma with 2 μM arsenic trioxide and 500 U/mL of IL-2, as an adjunct treatment for patients with refractory Acute Myeloid Leukemia (AML) undergoing reduced-intensity stem cell transplantation. The hypothesis was that pre-transplant administration of haploidentical NK cells could potentially reduce disease burden and result in improved outcomes after the transplant in patients with refractory AML (53). Arsenic trioxide (ATO) has been shown to increase the expression of NKG2D ligands, leading to enhanced NK cell recognition and killing of AML cells. Additionally, ATO can promote the activation and expansion of NK cells as well as enhance their cytotoxic function by modulating the expression of the activating and inhibitory receptors on NK cells. These mechanisms collectively contribute to the improved cytotoxicity of NK cells against AML cells (54).

A phase I clinical trial by Bednarski et al., 2019 (**Table 1**), demonstrated the feasibility and potential of this approach. An 18-month-old patient with relapsed AML received an infusion of donor-derived memory-like NK cells following FLAG chemotherapy and a T cell-based donor lymphocyte infusion (DLI). This resulted in complete remission and persistence of the memory-like NK cells for over 6 months, suggesting their potent anti-leukemic activity and prolonged *in vivo* persistence (56).

By overcoming these challenges and leveraging the advantages of allogeneic NK cells, this therapy has the potential to become a valuable tool in the fight against cancer, particularly for patients in whom autologous NK cell approaches are less effective.

#### 3.2.3 Cytokine stimulation and antibody-mediated activation

NK cells express receptors for several cytokines, including interleukin (IL)-2, IL-12, IL-15, IL-18, and IL-21, which allows rapid responses to inflammatory signals (8, 57). Additionally, one key receptor expressed on NK cells membrane is CD16 (FcγRIIIa), which is crucial in antibody-mediated activation. Optimizing NK cell activity through cytokine stimulation and antibody-mediated activation is a promising approach for cancer immunotherapy.

#### 3.2.4 Cytokine stimulation

Resting NK cells primarily express the intermediate-affinity IL-2 receptor (IL-2Rβγc) which can bind IL-2 but with a lower affinity compared to the high-affinity receptor (58, 59), while immature CD56bright NK cells constitutively express the high-affinity IL-2R (IL-2Rαβγ) (60). Mature CD56dim NK cells, in contrast, lack the high-affinity receptor. Upon activation, NK cells upregulate IL-2Rα expression, enhancing their sensitivity to IL-2 (22, 58). Notably, IL-2Rβ expression is more widespread among lymphocytes than IL-2Rα, with NK cells exhibiting the highest levels. Defects in IL-2Rα or IL-2Rβ can impede NK cell maturation and function, hindering the development of “memory-like” NK cells. The γc chain, while essential for IL-2 signaling, is less inducible compared to IL-2Rα and IL-2Rβ (60). Engagement of the intermediate-affinity IL-2 receptor promotes NK cell growth, cytotoxicity, and subsequent upregulation of IL-2Rα, thereby amplifying the IL-2 response. This activation also requires metabolic reprogramming, as demonstrated by reduced interferon-gamma (IFN-γ) and granzyme B (Gzmb) production when NK cells are cultured with glycolysis inhibitors during cytokine stimulation (61).

Cytokine signals also activate transcription factors, such as STATs and NFκB, leading to increased production of IFN-γ. IFN-γ plays a key role in the immune response by promoting MHC-I expression, differentiating T cells, and recruiting immune cells to the tumor microenvironment (TME) (25).

Studies by Pencheva-Demireva et al., 2019, studied upregulating NK cell proliferation through cytokine stimulation, specifically using IL-15 and IL-18. This combination effectively promoted NK cell proliferation and upregulated various activation receptors, suggesting their potential for cancer therapy. Further research is needed to evaluate the therapeutic efficacy of IL-15 and IL-18 stimulation *in vivo* (62).

Nevertheless, the use of cytokines to support allogeneic NK cell therapy holds promise but requires careful consideration. For example, a study by Berrien-Elliott et al., 2022, showed that systemic IL-15 administration in patients with relapsed AML led to accelerated rejection of donor NK cells by recipient CD8 T cells, highlighting the complex interplay among cytokines, host immunity, and NK cell persistence. Further research is needed to optimize cytokine protocols that balance NK cell expansion and minimize the risk of rejection (23).

#### 3.2.5 Antibody-mediated activation

The research by Duggan et al., 2018, focused on co-stimulating the Fc receptor (FcR) and interleukin-12 (IL-12) receptor on human NK cells. This co-stimulation increased the expression of CD25, a component of the high-affinity IL-2R. The study aimed to determine if stimulating NK cells via immobilized IgG and IL-12 could enhance CD25 expression and promote NK cell anti-tumor activity in response to low-dose IL-2 (63).

Their findings demonstrated that dual stimulation significantly enhanced CD25 expression compared to unstimulated cells or those treated with either IgG or IL-12 alone. Additionally, dual-stimulated NK cells displayed increased cytotoxicity against tumor cells and exhibited enhanced CD25 expression in patients with head and neck cancer following combination therapy. These findings suggest that FcR and IL-12R co-stimulation could be a potential strategy for maximizing NK cell activity in cancer treatment (63).

Probably, the most studied FcR of NK cells is CD16. CD16 receptors (FcγRIII) on NK cells play a crucial role in antibody-dependent cell-mediated cytotoxicity (ADCC). They bind to the Fc portion of specific antibody subclasses, triggering NK cell activation and target cell destruction.

Nakazawa et al., 2022, studied the use of antibodies targeting NKp46 and CD16 receptors for NK cell expansion. Anti-NKp46 stimulation increased NK cell purity and expansion compared to non-antibody-stimulated populations. Anti-CD16 stimulation also enhanced the expansion but to a lesser extent. Importantly, the expanded NK cells from all groups inhibited the growth of tumor cells, suggesting the enormous potential of this therapeutic approach (64).

Another example is Rezvani et al., 2021. They used AFM13, a bispecific antibody that targets both CD16A on natural killer (NK) cells and CD30 on tumor cells. This unique binding allows AFM13 to redirect NK cells towards CD30-positive tumor cells, enhancing their ability to recognize and kill these cancer cells. CD30 is a protein expressed on the surface of certain cancer cells, including lymphomas. By binding to CD30, AFM13 acts as a bridge between NK cells and tumor cells, facilitating their interaction and ultimately leading to tumor cell destruction.

To enhance the antitumor activity of NK cells, the researchers preactivated them with a combination of interleukin-12 (IL12), IL15, and IL18. This pre-activation step primed the NK cells, made them more responsive to AFM13 and improved their ability to kill tumor cells (65, 66). The study found that preactivated NK cells loaded with AFM13 demonstrated a superior antitumor activity compared to NK cells without pre-activation. *In vivo* experiments confirmed the safety and efficacy of this approach, with AFM13-loaded NK cells effectively targeting and killing CD30-positive lymphoma cells without causing significant toxicity. These findings suggest that combining AFM13 with preactivated NK cells could be a promising strategy for treating CD30-positive lymphomas (66).

**Figure 2:**
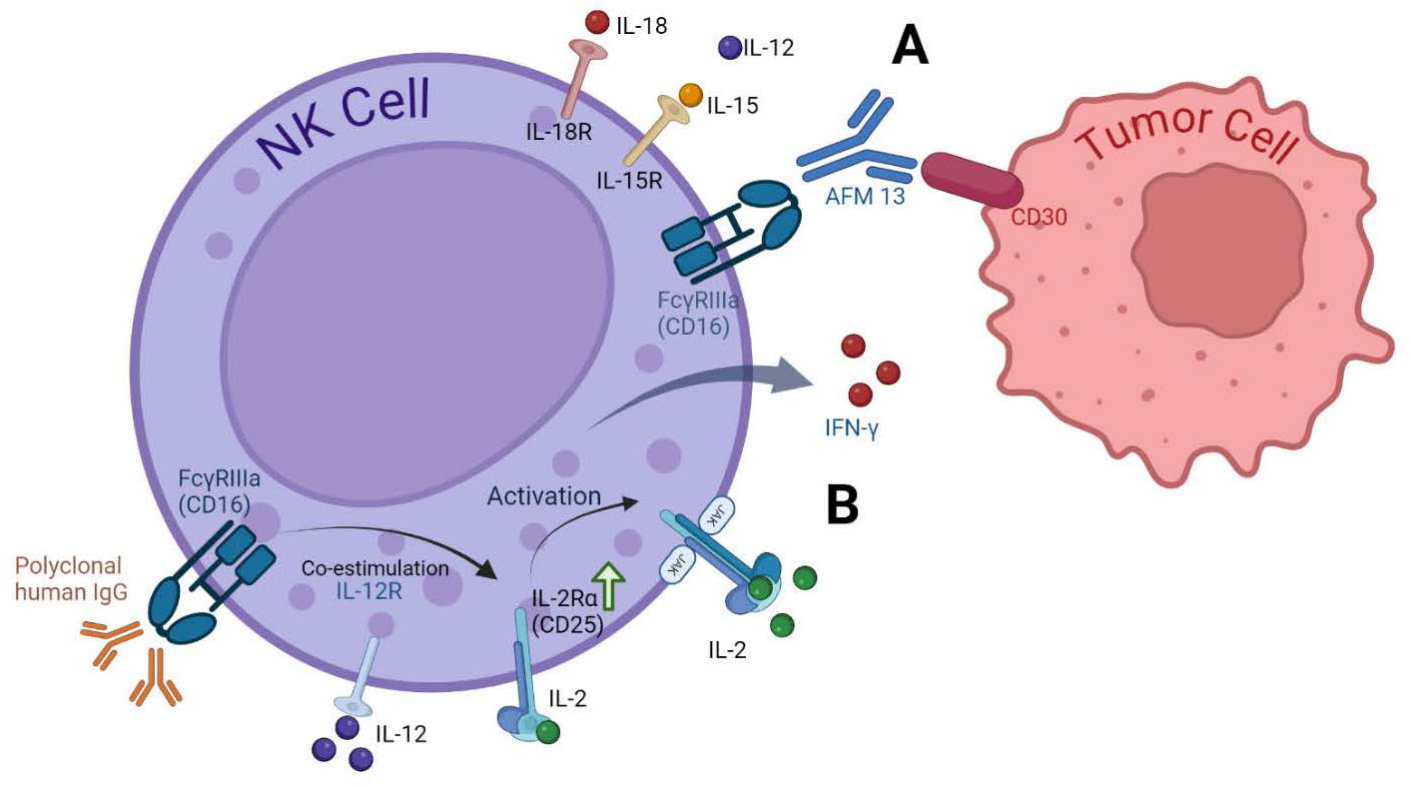
Cytokine receptors on NK cells and activation strategies. **(A)** Enhancing NK Cell Anti-Tumor activity with AFM13. The bispecific antibody AFM13 effectively activates NK cells and enhances their ability to recognize and kill CD30-positive tumor cells. Pre-activating NK cells with IL12/15/18 further amplifies these anti-tumor effects (66). **(B)** Dual Stimulation with IgG and IL-12. Stimulating NK cells with both immobilized IgG and IL-12 significantly upregulates CD25 expression, a component of the high-affinity IL-2 receptor. This increased CD25 expression leads to greater responsiveness to low-dose IL-2, resulting in enhanced NK cell signaling, cytokine production (IFN-γ), and cytotoxicity. This approach has shown effectiveness in clinical settings, as demonstrated by increased CD25 expression in head and neck cancer patients receiving cetuximab and IL-12 therapy (63).

By stimulating NK cell activity through cytokines and specific antibodies, researchers are developing novel strategies to harness the potential of NK cells for cancer immunotherapy. Optimizing these activation methods and combining them with other therapeutic approaches holds promise for improving the efficacy of NK cell-based cancer treatments **Figure 2**.

#### 3.2.6 Targeting specificity with engineered receptors: CAR-NK cells

Chimeric antigen receptor T-cell (CAR-T) therapy has revolutionized cancer treatment by demonstrating its efficacy against various malignancies, including gastric cancer (67), glioblastoma (68), multiple myeloma (69), B-cell non-Hodgkin lymphoma (70), acute lymphoblastic leukemia (ALL) (71, 72), and many others. However, CAR-T therapy faces significant limitations:

**Individualized Manufacturing:** Current approaches rely on autologous CAR-T cells, requiring personalized production for each patient, which is time-consuming and very expensive (73).

**Graft-Versus-Host Disease (GVHD):** Allogeneic CAR-T cells carry a risk of GVHD, a serious complication where the donor’s immune cells attack the recipient’s body (73).

**Relapse and Toxicity:** A significant portion of patients experience disease recurrence after CAR-T therapy, and these treatments can cause cytokine release syndrome (CRS) and neurotoxicity (73, 74).

To overcome these limitations, and trying to reach a better recognition and lower cytotoxicity, different immune cells including γδ T lymphocytes (75), Vα24-invariant natural killer T cells (NKTs) (76), Natural Killer cells (NK) (77) and even macrophages (78) are engineered to express chimeric antigen receptors (CARs) (9); specifically, CAR-NK cells offer several advantages over CAR-T cells:

**Reduced Risk of GVHD:** Unlike T cells, NK cells have a lower risk of causing GVHD when they are derived from allogeneic donors (10). Clinical trials have shown that CAR-NK cells can achieve high response rates without major toxic effects (**Table 1**), indicating a favorable safety profile (77, 79, 80). Also, CAR-NK cells exhibit minimal or no cytokine release syndrome (CRS) and neurotoxicity, which are common complications as we mentioned before in CAR-T cell therapies.

**Shorter Lifespan:** NK cells have a shorter lifespan in the bloodstream than T cells (81), potentially reducing the risk of long-term toxicities. The limited expansion and replicative senescence contribute to the short *in vivo* persistence of NK cells. This allows a greater control in the immune response of the cells within the host, which could be considered an advantage by further reducing their potential for adverse effects in the long term (9, 82, 83). In any case, increasing the lifespan of the cells would make their use more efficient and reduce their production cost, which is why today the use of Car-T cells is still a better option.

**“Off-the-Shelf” Potential:** Allogeneic CAR-NK cells can be derived from healthy donors and cryopreserved, creating a readily available “off-the-shelf” cell therapy product. This allows for immediate availability and broader application (79, 84, 85).

Clinical trials like the one by Liu et al., 2020, demonstrated the promise of CAR-NK therapy. This study showed that allogeneic CAR-NK cells targeting CD19 were well-tolerated and effective in patients with relapsed or refractory CD19-positive cancers, highlighting the potential of CAR-NK as an alternative immunotherapy approach (52, 77).

Several research efforts are underway to improve CAR-NK cell therapy. Some of them include:

**CAR Design:** Engineering CARs with specific co-stimulatory domains (like the transmembrane domain of NKG2D or the 2B4 co-stimulatory domain) and signaling pathways (like CD3ζ signaling domain) can enhance NK cell activity against tumors (86). Another example is the research of Chockley et al., 2023; they studied the use of a PDZ binding motif (PDZbm) to enhance CAR synapse formation and cell polarization in NK cells. The PDZbm was chosen because it has been associated with synapse formation and polarity of immune cells, and the researchers aimed to modulate or tune the synapse to an increasingly ordered state by adding it to the CAR. This improved design resulted in an increased effector cell functionality, cytokine secretion, tumor cell killing, and prolonged survival in animal models. This research highlights the potential of CAR engineering and synapse modulation to improve the efficacy of CAR-NK cell therapy for solid tumors (87).

**NK Cell Source:** Different sources of NK cells are being explored, including induced pluripotent stem cells (iPSCs), which offer advantages like standardized cell populations and large-scale production (86).

**Delivery Methods:** Non-viral methods like mRNA-lipid nanoparticles (LNP) are being studied for safe and efficient CAR introduction into NK cells (88).

On the other hand, the production of CAR-NK cells involves a multi-step process which makes its manufacture not so easy:

**NK Cell Source Selection:** Choosing a source of NK cells, such as PBMC-derived NK cells, NK cell lines, cord blood, hPSC-derived NK cells, or iPSC-derived NK cells.

**NK Cell Stimulation and Enrichment:** Enriching NK cells using methods like CD56+ beads and stimulating them to improve their function.

**CAR Transfection:** Introducing the CAR gene sequence using viral vectors or non-viral methods.

**NK Cell Expansion and Activation**: Expanding the CAR-NK cells using cytokines (IL-2, 15, 18, 21, and 12) and activating them for optimal anti-tumor activity.

**Cryopreservation and Quality Control:** Freezing CAR-NK cells for storage and performing quality checks to ensure CAR expression and functionality.

**Preconditioning and Administration:** In some cases, patients may undergo chemotherapy or bone marrow depletion to improve the effectiveness of CAR-NK therapy (89).

However, CAR-NK cell therapy holds an immense potential as a safer and more readily available approach for cancer immunotherapy. By optimizing CAR design, NK cell source, and manufacturing processes, researchers are paving the way for this promising new treatment strategy.

## 4 Influence of the microenvironment on efficacy of NK cell-based therapies in solid tumors and hematological malignancies

The efficacy of NK cell-based therapies is significantly hampered by the immunosuppressive tumor microenvironment (TME) (90). This section explores the multifaceted mechanisms by which TME disrupts NK cell metabolism and function, ultimately limiting their antitumor activity. Additionally, we discuss emerging therapeutic strategies in both cases, solid tumors and hematological diseases.

### 4.1 Metabolic reprogramming and nutrient deprivation

The TME is characterized by a metabolic shift towards aerobic glycolysis, leading to competition for glucose and other vital nutrients between tumor cells and infiltrating immune cells (24). This nutrient scarcity significantly impacts NK cell function. Studies have demonstrated that tumors deprive NK cells of essential amino acids such as arginine, leucine, and glutamine, all crucial for supporting their proliferation and effector functions (26, 91). Furthermore, glucose restriction disrupts the mammalian target of rapamycin complex 1 (mTORC1) signaling pathway, a key regulator of NK cell metabolism. IL-15 activation of mTORC1 via the PI3K-PDPK1-AKT pathway upregulates glycolysis and enhances NK cell function (24). However, the nutrient-depleted TME likely disrupts this finely tuned metabolic regulation, hindering the NK cell effector activity.

Hypoxia, another defining feature of the TME, further compromises NK cell function. Low oxygen levels, along with the influence of hypoxia-inducible factor 1 alpha (HIF1α) and oxidative stress pathways, contribute to mitochondrial dysfunction and morphological alterations in tumor-infiltrating NK cells. This not only disrupts the cellular energy production but also weakens the overall NK cell function and cytotoxic activity (90).

### 4.2 Immunosuppressive metabolites and cytokines

The TME accumulates a plethora of immunosuppressive metabolites that directly target and suppress NK cells. Adenosine, a byproduct of tumor metabolism, plays a particularly detrimental role by inhibiting NK cell proliferation and effector functions (26). Additionally, lactate, another key metabolite produced by tumor cells due to their reliance on aerobic glycolysis, specifically suppresses NK cell cytotoxicity by inhibiting IFNγ production and NFAT activation (24, 92).

On the other hand, TME is often enriched with anti-inflammatory cytokines such as TGF-β, which can potently suppress NK cell activity. This effect is particularly pronounced in tumors of the breast and liver. The presence of TGF-β further dampens NK cell function and effector responses, creating a complex and immunosuppressive environment that obstructs their antitumor potential (90).

### 4.3 Solid tumors: progress and challenges in different cancer types

Researchers are making significant strides in enhancing NK cell targeting and activation to improve their effectiveness against solid tumors. Here are some promising strategies:

#### 4.3.1 Engineering for specificity

Scientists are developing methods to equip NK cells with enhanced tumor-targeting capabilities. This is the case of glycoengineering, where Gong et al. decorated NK cells with nanobodies, which are small antibody fragments that can bind to specific targets. In this case, they used the 7D12 nanobody which is a single-domain antibody derived from camelid heavy-chain-only antibodies. It specifically targets the human epidermal growth factor receptor (EGFR), which is overexpressed in many types of cancers. This nanobody has been explored for various applications in cancer diagnosis, imaging, and therapy due to its high specificity and affinity for EGFR. The 7D12 nanobody was chemically modified with a DBCO group, a molecule that can react with azide groups through click chemistry. Simultaneously, NK92MI cells were engineered to express azide groups on their surface. By utilizing click chemistry, the DBCO-modified nanobody was attached to the azide-modified NK cells, resulting in the creation of 7D12-NK92MI cells.

The modified NK cells had increased cytokine secretion; they preferentially targeted and killed tumor cells that overexpressed EGFR in a wide variety of tumor types, and they could infiltrate tumors effectively apparently with minimal side effects (93).

#### 4.3.2 Harnessing existing mechanisms

Researchers are exploring ways to leverage existing NK cell functions to improve their anti-tumor activity. One strategy focuses on enhancing Antibody-Dependent Cellular Cytotoxicity (ADCC), a process where NK cells destroy antibody-coated tumor cells (6). Additionally, the immunological synapse (IS), the contact point between an NK cell and a target cell, is being researched for targeted drug delivery directly to the tumor site.

In the study of Im et al., 2020, they tried to develop a more effective cancer therapy by combining the natural killing ability of NK cells with targeted drug delivery. The researchers focused on the immunological synapse (IS), a structure formed when NK cells interact with cancer cells, as a potential trigger for targeted drug delivery. A micellar system, composed of the hydrophilic polymer poly (ethylene glycol) and the hydrophobic polymer poly (β-aminoester), was chemically modified to encapsulate doxorubicin (DOX), a widely used chemotherapeutic drug. This micellar system was then conjugated to NK cells, forming reinforced NK cells (ReNK), through a maleimide-thiol coupling reaction.

The researchers observed that the acidic environment generated at the IS during NK cell-mediated tumor killing triggered the disassembly of the micellar system, leading to the release of DOX. This localized drug release was confirmed through confocal microscopy, which visualized the colocalization of DOX with the tumor cells. *In vitro* and *in vivo* studies demonstrated the superior antitumor efficacy of ReNK compared to traditional chemotherapy. ReNK-treated mice exhibited significantly reduced tumor growth and prolonged survival, while exhibiting minimal systemic toxicity (94).

This novel approach, which takes advantage of the natural ability of NK cells to localize tumors and IS-dependent drug release, offers a promising strategy to develop more effective and less toxic cancer treatments. By targeting drug delivery to the tumor site, ReNK could potentially reduce the adverse side effects associated with traditional chemotherapy, as well as increase the lifespan of NK cells.

The nature of NK cells already provide many advantages in cancer treatment strategies, but the tumor microenvironment must always be considered. Shaim et al., 2021. investigated the potential of natural killer (NK) cell-based immunotherapy for treating glioblastoma multiforme (GBM), a highly aggressive brain cancer. The study found that NK cells can infiltrate GBM tumors but often exhibit an impaired function due to the immunosuppressive effects of glioblastoma stem cells (GSCs). GSCs release transforming growth factor-beta (TGF-β), a potent cytokine that disrupts NK cell activity (95, 96). By targeting TGF-β signaling using specific inhibitors, such as galunisertib and LY2109761, researchers were able to restore NK cell function and enhance their ability to kill GSCs. These findings suggest that combining NK cell therapy with TGF-β inhibitors may be a promising strategy to improve GBM treatment outcomes (97).

#### 4.3.3 Novel delivery techniques

Innovative methods are being explored to improve the NK cell delivery and infiltration into tumors. One approach utilizes magnetic nanoparticles conjugated to antibodies that recognize NK cells. This allows researchers to guide the NK cells directly to the tumor site using an external magnetic field (98).

Another exciting development involves equipping NK cells with aptamers, which are short, single-stranded DNA or RNA molecules that can bind to specific target molecules with high affinity and specificity, similar to antibodies. These aptamers can be designed to bind specifically to tumor cells and potentially block immune checkpoints, enhancing the NK cell activity within the tumor microenvironment. In this study, aptamers were chemically equipped to NK cells to specifically target tumor cells and enhance their immunotherapeutic potential. The strategy offers a simple and safe technology to engineer NK cells with active tumor targeting, deep penetration, and a high therapeutic efficiency, holding considerable potential for adoptive immunotherapy in solid tumors (99).

There is also the possibility to combine CAR design to enhance delivery and infiltration of NK cells into tumors using them as drug delivery vehicles. These engineered NK cells are designed to target specific tumor antigens and deliver anticancer drugs directly to the tumor site. To evaluate the efficacy and tumor specificity of these cells, researchers developed a time-lapse imaging method using two-photon microscopy in both live mouse models and 3D tissue cultures.

A key innovation of this research is the development of a 3D tissue culture model that more accurately replicates the complex tumor microenvironment. This model enables a more realistic assessment of CAR-NK cell behavior and efficacy. Additionally, the use of intravital two-photon microscopy allows for real-time visualization of cell trafficking, infiltration, and drug delivery within living organisms.

The novel aspect of this research lies in the development of a strategy that uses living CAR-NK cells as carriers to deliver anticancer drugs specifically to the tumor site. This approach addresses the challenge of overcoming complex barriers within the tumor microenvironment (TME) that hinder the efficacy and trafficking of CAR-NK cells in solid tumors. The study further introduces a time-lapsed method to evaluate the efficacy and tumor specificity of CAR-NK cells using two-photon microscopy in live mouse models and 3D tissue slide cultures.

Photosensitive drugs, such as benzoporphyrin derivative (BPD), function as photosensitizers with photodynamic and photothermal efficacy. When activated by light (in this study, a 680 nm laser), these drugs induce cytotoxic effects on target cells. The study demonstrates that the combined use of CAR-NK cells and photosensitive chemicals, such as BPD, enhances antitumor immunity in both *in vitro* and *in vivo* tumor models. Moreover, the study visualizes the trafficking, infiltration, and accumulation of drug-loaded CAR-NK cells within deeply situated tumor microenvironments using non-invasive intravital two-photon microscopy. This research highlights the potential of this approach for combined cellular and small-molecule therapies in cancer treatment (100).

Early clinical studies have yielded promising results regarding the safety and efficacy of NK cell therapies. For instance, a study by Khatua et al., 2020, demonstrated the feasibility and safety of NK cell infusions in treating children with recurrent medulloblastoma and ependymoma (52). While the therapeutic effect requires further research, the study paves the way for further clinical trials in solid tumors (6, 101–105).

### 4.4 Hematological malignancies: promising results in leukemias and lymphomas

NK cells offer a promising path for treating hematological malignancies. Significant progress has been made in overcoming limitations and improving the effectiveness of NK cell-based immunotherapy. However, there are still challenges that need to be addressed.

In the same way, there are studies that have shown excellent progress in the use of these cells in the treatment of leukemias and lymphomas. Studies by Szmania et al., 2015, demonstrated the safety and feasibility of infusing expanded autologous or haploidentical NK cells in patients with high-risk relapsed myeloma. This approach resulted in significant *in vivo* expansion of NK cells and no serious adverse effects (50). Tomaipitinca et al., 2021, discussed strategies to improve NK cell trafficking and localization (106). Here, we describe some of the most novel approaches enhancing NK cell-based immunotherapies in hematological malignances.

#### 4.4.1 Bi-specific and Tri-specific Killer cell Engagers

These engineered molecules bridge the gap between NK cells and tumor cells, promoting an efficient immune synapse formation. BiKEs and TriKEs consist of 2 or 3 single-chain variable fragments (scFv) that bind to NK cell receptors (e.g., CD16) and tumor-associated antigens (TAAs) (107). Compared to monoclonal antibodies, BiKEs and TriKEs offer advantages such as a smaller size, improved biodistribution, and lower immunogenicity. The study by Sivori et al., 2021, highlights the potential of BiKEs and TriKEs, particularly those targeting CD16 on NK cells and TAAs such as CD33, EpCAM, and CD133 on tumor cells (107). But there is a variety of these antibodies used in clinical trials for different targets. TriKEs, such as the 161519 TriKE (anti-CD16, IL-15, and anti-CD19), have been shown to significantly enhance NK cell proliferation, activation, cytokine secretion, and cytotoxicity against CD19+ tumor cells, leading to an improved tumor growth inhibition and prolonged survival in preclinical models of B-cell lymphoma (108).

Nowadays, BiKEs and TriKEs are being studied for their potential in treating various types of leukemia, such as acute myeloid leukemia (AML), acute lymphoblastic leukemia (ALL), chronic myeloid leukemia (CML), and chronic lymphocytic leukemia (CLL) (109). Additionally, the incorporation of IL-15 within TriKEs is being explored to enhance NK cell activation and persistence (110, 111).

#### 4.4.2 Metabolic rejuvenation

Pre-activating NK cells with IL-15 *ex vivo* before infusion can enhance their metabolic fitness and antitumor activity by upregulating glycolysis and other metabolic pathways (24). Additionally, manipulating the TME with specific inhibitors and stimulants that regulate nutrient availability and metabolic pathways in NK cells holds promise to improve their function within the tumor environment.

#### 4.4.3 CAR-NK cells

As discussed in section 3, engineering NK cells with chimeric antigen receptors (CARs) allows them to directly target tumor cells, bypassing some of the suppressive mechanisms of the TME, such as antigen presentation dependence.

Nanoparticle-Based Delivery Systems: Nanoparticles offer a unique platform for NK cell therapy. They can be used to deliver therapeutic agents such as cytokines, genes, or antibodies directly to NK cells within the body, promoting their activation and expansion *in vivo*. Additionally, nanoparticles can be used to enhance NK cell engagement with tumor cells by improving targeting and delivery strategies (112). Targeting Immunosuppressive Metabolites: Strategies to target immunosuppressive metabolites as adenosine in the TME are being explored as a potential approach to improve NK cell activity and overcome the suppressive effects of the tumor environment (24). Monoclonal antibodies targeting NK cell checkpoints (e.g., PD-1/PD-L1) and other inhibitory receptors can reverse NK cell exhaustion and improve their anti-tumor activity. These therapies have demonstrated clinical benefits in hematological cancers (113, 114). Ectoenzymes are another important research topic in this field. Ectoenzymes are enzymes located on the cell surface that catalyze biochemical reactions outside the cell. The CD73, CD39, and CD38 ectoenzymes are involved in the extracellular production of adenosine, a potent immunosuppressive metabolite. Adenosine can inhibit the function of natural killer (NK) cells, thereby impairing their anti-tumor activity. Targeting these ectoenzymes and their associated pathways represents a promising strategy to augment the efficacy of NK cell-based immunotherapies (115–117).

## 5 Challenges and considerations

Natural killer (NK) cells have emerged as promising candidates for cancer immunotherapy due to their ability to directly recognize and kill tumor cells. However, several challenges and considerations remain to be addressed before NK cell-based therapies can be widely translated into clinical practice (118).

### 5.1 Allogeneic NK cell therapy

While allogeneic NK cells have shown potential in clinical trials in hematological malignancies with the risk of graft-versus-host disease (GVHD) there remains a significant concern. Efforts are underway to develop strategies to prevent or manage GVHD in allogeneic NK cell therapy, such as strategies to eliminate contaminating T cells, optimize donor selection, use immunosuppressive agents, and engineer NK cells to reduce their alloreactivity (7, 10, 83, 118).

**Sourcing NK Cells**: Identifying the optimal source of NK cells for therapy is crucial. Different sources such as blood, umbilical cord blood, and stem cells offer advantages and limitations in terms of functionality, availability, and potential for Graft-versus-Host Disease (GvHD) (6).

**Solid Tumor Microenvironment**: The solid tumor microenvironment is often immunosuppressive, and hinders the NK cell activity and persistence within the tumor. Iannone et al., 2015, highlighted the importance of understanding how the tumor microenvironment affects NK cell activity. Their findings suggest that surgery and the tumor stage can influence the number and functionality of NK cells within the tumor, impacting their ability to eliminate residual cancer cells (101). Nowadays researchers are actively investigating strategies to overcome these suppressive mechanisms and enhance NK cell function within the tumor (6, 102).

**GvHD**: The use of allogeneic NK cells derived from another donor carries a risk of GvHD, a serious condition where the donor’s immune system attacks the recipient’s body (6). Mitigating this risk is crucial for the safe and widespread use of allogeneic NK cell therapy.

Further research is essential to fully unlock the potential of NK cell-based immunotherapy for solid tumors. This includes: Optimizing methods for NK cell expansion and maintaining their functionality *ex vivo*, developing strategies to enhance NK cell persistence and overcome the immunosuppressive nature of the tumor microenvironment, and minimizing the risk of GvHD is associated with allogeneic NK cell therapy (102).

Also, MDSCs (myeloid-derived suppressor cells), a heterogeneous population of immature myeloid cells, play a crucial role in promoting tumor progression, metastasis, and creating an immunosuppressive tumor microenvironment (TME). MDSCs are characterized by their ability to suppress immune responses, particularly by inducing T-cell and natural killer (NK) cell anergy through various mechanisms, including the expression of enzymes such as IDO, ARG1, iNOS, and NOX2 (104). To limit MDSC in the TME for NK cell-based immunotherapy, one strategy is to target the CCR5 chemokine receptor on MDSCs to inhibit their recruitment and immunosuppressive activity. Another approach is to disrupt CXCR2-mediated migration of MDSCs to the TME, which can significantly improve anti-PD1 treatments. Additionally, combining checkpoint inhibitors with MDSC depletion using epigenetic modulatory drugs such as entinostat and 5-azacytidine has shown promising results in complete tumor regression and improved survival rates in aggressive cancer models (105).

By addressing these challenges and building upon the exciting progress in NK cell engineering and delivery methods, NK cell-based immunotherapy holds significant promise for improved clinical outcomes in patients with solid tumors.

### 5.2 Enhancing NK cell function

Cytokines, such as interleukin-15 (IL-15) and interleukin-21 (IL-21), can effectively enhance NK cell function, but they can also cause significant side effects, including fever, fatigue, and flu-like symptoms (119). Moreover, tumor cells can develop resistance to cytokine-induced NK cell activation, limiting the effectiveness of this approach (120). Strategies to overcome these challenges include developing novel cytokine analogs with reduced side effects, combining cytokines with other immunomodulatory agents, and targeting specific signaling pathways involved in NK cell activation (118, 119, 121).

### 5.3 Targeted therapies

Therapeutic antibodies and bispecific/trispecific killer engagers (BiKEs/TriKEs) can be used to target and activate NK cells against specific tumor antigens. However, these agents can also bind to unintended targets leading to off-target effects and reduced efficacy. Additionally, tumor cells can develop resistance to targeted therapies and hematological conditions often involve immune dysfunctions that hamper effective anti-tumor responses (122, 123). To address these challenges, researchers are exploring the development of more specific targeting molecules, combination therapies with other agents, and strategies to overcome tumor resistance. Definitely, making personalized approaches is necessary to face these challenges (114, 120).

### 5.4 CAR-NK cells

Chimeric antigen receptor (CAR)-expressing NK cells have shown promising preclinical and clinical results in hematological malignancies. However, the production of CAR-NK cells can be complex and expensive, which limits their availability for clinical use. Furthermore, CAR-NK cells may not persist or function optimally within the tumor microenvironment, leading to suboptimal therapeutic responses (82, 83). Efforts are being made to improve the manufacturing and persistence of CAR (112, 122, 123)-NK cells, including optimizing culture conditions, engineering CAR-NK cells to express survival factors, and combining them with other immunomodulatory agents (124–126).

Some groups have sought to address these problems from a genetic approach. For example, the studies by Huang et al., 2021, explored a non-viral approach for engineering NK cells using CRISPR-Cas9 ribonucleoproteins (Cas9 RNPs). This method enabled efficient gene editing, including multiplex knockout, in-frame gene knock-in, and *ex vivo* expansion of cryopreserved primary human NK cells. This research offers a robust and versatile platform for engineering NK cells without the limitations associated with viral vectors or plasmid transfection (103).

### 5.5 Optimizing NK cell expansion and function

**Figure 3:**
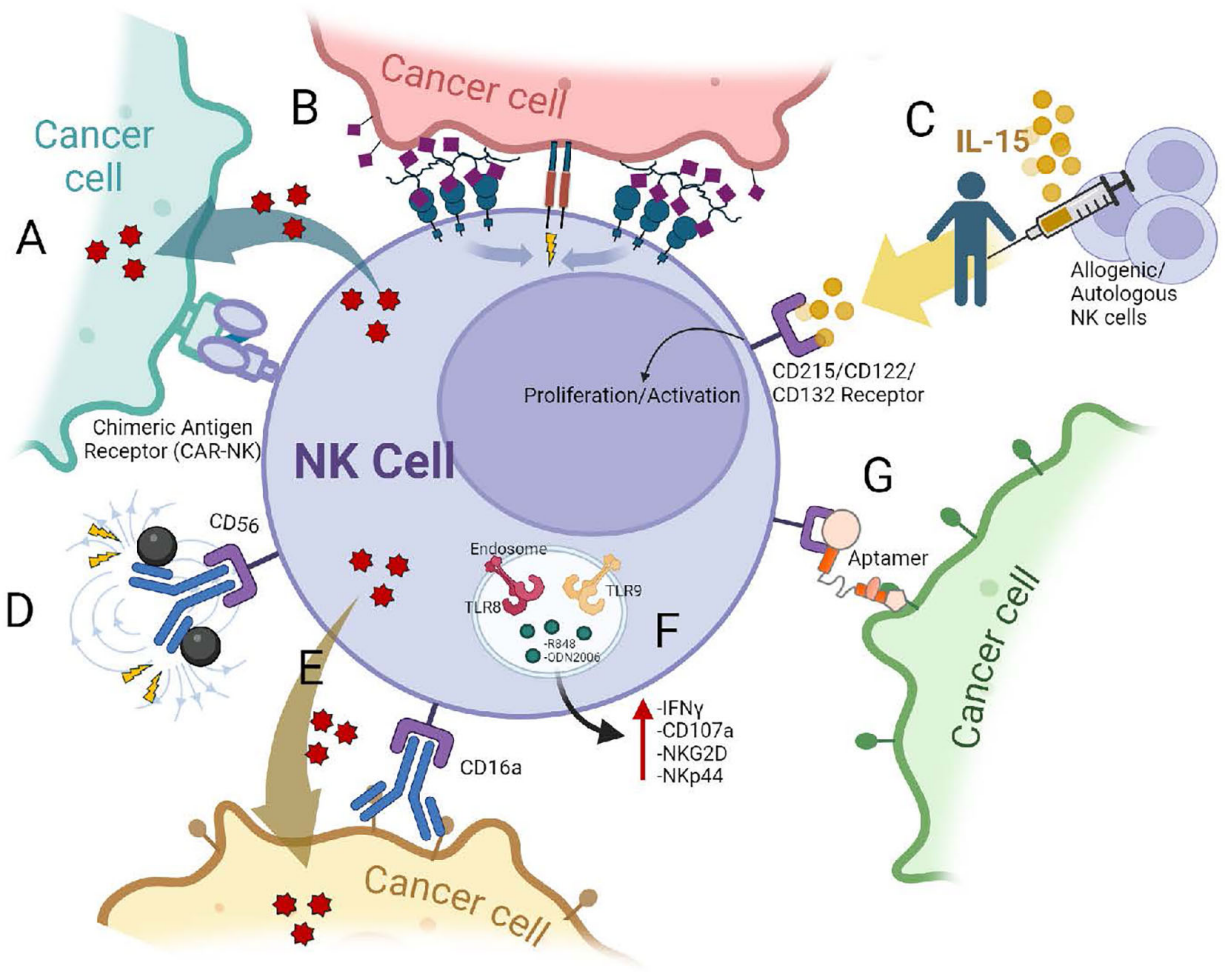
Some strategies to enhance the cytotoxic functions of NK cells against cancer cells: **(A)** NK cells expressing Chimeric Antigen Receptors (CARs). CARs with specific co-stimulatory domains, such as the NKG2D transmembrane domain, can enhance NK cell activity against tumors. **(B)** Glycoengineering. This approach equips NK cells with enhanced tumor-targeting capabilities. Cells are decorated with molecules (nanobodies) that specifically recognize and bind to tumor cells (93). **(C)** Cytokine Stimulation. IL-15 signals through a heterotrimeric receptor consisting of IL-15Rα (CD215), IL-2/IL-15Rβ (CD122), and the common γ chain (γch, CD132) (128). Similar to IL-2 or IL-18, stimulation of NK cells with these cytokines increases their proliferation rate and cytotoxic activity. **(D)** Magnetic nanoparticles conjugated to antibodies that recognize NK cells. Anti-CD56 antibodies conjugated with Fe3O4 nanoparticles allowing for specific binding with NK cells and endowing them with magnetic field-driven targeting ability (98). **(E)** Enhancing Antibody-Dependent Cellular Cytotoxicity (ADCC). While NK cells destroy antibody-coated tumor cells through its FcγRIIIa (CD16), antibodies targeting NKp46 and CD16 receptors on NK cells can also promote cell expansion. **(F)** Endosomal TLR activation. TLR stimulation significantly enhances NK cell activation and cytotoxicity against leukemic cells, with TLR8 and TLR9 ligands showing particular promise as potential antitumor therapies (33). **(G)** Aptamer Technology. This technology utilizes small, engineered RNA or DNA molecules (aptamers) designed to bind specifically to tumor cells. These aptamers have the potential to block immune checkpoints, enhancing NK cell activity within the tumor microenvironment (99).

The *ex vivo* expansion of NK cells is essential for clinical applications, but current methods can be challenging and may not preserve their full functional capacity. Additionally, maintaining the desired phenotype and function of NK cells during expansion can be difficult. Research is ongoing to develop improved culture conditions, optimize the use of cytokines and growth factors, and explore genetic engineering approaches to enhance NK cell expansion and function (114, 125, 127) **Figure 3**.

## 6 Discussion

### 6.1 Summary of key findings and future potential of NK Cell-based immunotherapies

The review highlights NK cells, immune system soldiers that eliminate virally-infected or cancerous cells without prior antigen exposure. They identify stressed or transformed cells through activating receptors, as NKG2D, that recognize ligands such as MICA/B and ULBPs; however, inhibitory receptors prevent NK cells from attacking healthy tissues. Cancer cells may exploit this by downregulating MHC-I, a self-recognition molecule, to evade immune attack.

It is evident that a key point is the sourcing of NK cells. They can be autologous (from the patient) - safer but potentially limited in availability or quality - or allogeneic (from a donor) - readily available and diverse but requiring matching and immunosuppression to minimize rejection. *Ex vivo* techniques can expand and activate NK cells before infusion using cytokines, co-culturing with stimulatory cells, or genetically modified feeder cells. Additionally, cytokine stimulation and antibodies targeting stimulatory receptors can further enhance NK cell function.

A particularly exciting approach involves engineering NK cells with CARs (chimeric antigen receptors) for a superior target recognition and lower cytotoxicity. This “CAR-NK” therapy offers advantages over CAR-T therapy, including potentially lower risk of graft-versus-host disease (GVHD) and the possibility of “off-the-shelf” treatments.

The immunosuppressive tumor microenvironment (TME) significantly hinders NK cell function. The TME disrupts NK cell metabolism and produces immunosuppressive factors. Additionally, optimizing methods for large-scale production of high-purity, functional NK cells remain crucial

Despite these hurdles, NK cell-based immunotherapy holds immense potential. Researchers are developing strategies to enhance NK cell targeting, activation, and persistence within the tumor microenvironment. This approach shows promise for treating a wide range of cancers, particularly hematological malignancies such as AML and MM. The future is bright for NK cell therapy, with CAR-NK cells and strategies to overcome the TME offering exciting possibilities for cancer patients.

### 6.2 Looking ahead: continued innovation and advancement in this promising field

Optimizing methods for sourcing, expanding, and activating NK cells remains a top priority. Researchers are also designing next-generation CARs to further enhance NK cell function and target specificity. However, addressing the complexities of large-scale CAR-NK cell manufacturing is crucial to make this therapy more readily available to patients.

The immunosuppressive tumor microenvironment (TME) presents a significant obstacle. Can we metabolically engineer NK cells or manipulate the TME itself to improve their effectiveness? CAR-NK cells and BiKEs/TriKEs (engineered NK cells with additional activating receptors) offer potential to overcome TME suppression, but safety concerns regarding these engineered therapies need a careful evaluation.

Non-viral gene editing techniques hold advantages for NK cell engineering; however, ethical considerations surrounding their use must be addressed. Additionally, understanding the mechanisms behind the up-regulation of inhibitory receptors on NK cells and the down-regulation of ligands on tumor cells in cancer patients is crucial. This knowledge could help reverse autologous NK cell deficiency, a common observation in such patients.

Further research is necessary to explore the therapeutic potential and safety of allogeneic NK cells, KIR-blocking antibodies (which enhance NK cell recognition of tumors), and NK cell-boosting cytokines such as IL-15 and IL-21. These alternative strategies hold promise for patients with hematological malignancies, such as AML, ALL, and MM.

Another exciting avenue lies in studying signaling molecules that limit NK cell activation across different activating receptors. By targeting and modulating these molecules, we can potentially enhance NK cell reactivity against cancer. This could involve a combination of strategies such as promoting NK cell activation and sensitizing tumor cells to NK cell-mediated killing through the modulation of apoptosis.

In any case, the field of NK cell immunotherapy is rapidly evolving. By addressing the challenges and exploring these promising avenues, we can unlock the full potential of NK cells to revolutionize cancer treatments. The research and outcomes presented in this review provide a foundation for proposing innovative therapeutic combinations. The true challenge lies in determining the optimal way to integrate these diverse approaches to harness their individual advancements synergistically.

Synergistic approaches combining NK cell-based immunotherapies with other treatment modalities offer a promising avenue to enhance cancer treatment efficacy while minimizing adverse effects. NK cell-based therapies, including adoptive NK cell transfer, CAR NK cells, and NK cell engagers, have demonstrated a significant potential in clinical trials. When combined with cytokine-based therapies or monoclonal antibodies, these strategies can potentiate the cytotoxic activity of NK cells against tumor cells.

Furthermore, integrating NK cell-based immunotherapies with traditional treatments like chemotherapy and radiation can create a multi-pronged approach, targeting cancer cells through diverse mechanisms and potentially improving overall outcomes. By carefully selecting and combining these therapeutic approaches, we aim to optimize the treatment responses and achieve complete remission in cancer patients while minimizing toxicity.

As we move forward, the emphasis should be on rigorous clinical trials to evaluate the safety and effectiveness of these combinations. Understanding the interactions between different immunotherapeutic agents and their effects on the immune system will be crucial. The ultimate goal is to develop personalized treatment regimens that leverage the strengths of each therapy, leading to better patient outcomes and a higher quality of life for cancer survivors.

## 7 Conclusion and future research directions

NK cells are emerging as powerful weapons in the fight against cancer. Their inherent ability to recognize and eliminate tumor cells makes them ideal candidates for immunotherapy. Advancements in sourcing, expanding, and engineering NK cells, particularly with CARs, are fueling the development of this novel therapeutic approach. While challenges remain, such as optimizing large-scale NK cell production and overcoming the immunosuppressive tumor microenvironment, researchers are actively developing solutions. By leveraging these advancements, NK cell-based therapies have the exciting potential to become a valuable tool in the fight against a wide range of cancers.

To overcome the challenges and limitations of NK cell-based immunotherapy, future research should focus on: developing strategies to prevent or manage GVHD in allogeneic NK cell therapy, identifying novel cytokine analogs with reduced side effects, overcoming tumor resistance to targeted therapies and cytokine stimulation, improving the manufacturing and persistence of CAR-NK cells, enhancing NK cell function within the tumor microenvironment, and exploring combination therapies with other immunomodulatory agents.

By addressing these challenges and continuing to explore innovative approaches, NK cell-based immunotherapy holds significant promise to improve outcomes in patients with hematological malignancies and other types of cancer.
